# Nutritional practices in long-term care across five European countries: Findings from the COST Action PROGRAMMING

**DOI:** 10.1016/j.jnha.2025.100650

**Published:** 2025-08-26

**Authors:** Anna Rudzińska, Gulistan Bahat, Gülçin Özalp, Serdar Özkök, Sumru Savas, Biljana Petreska-Zovic, André Rodrigues, Evrydiki Kravvariti, Anne Wissendorff Ekdahl, Sofia Duque, Mirko Petrovic, Marina Kotsani, Karolina Piotrowicz

**Affiliations:** a Department of Internal Medicine and Gerontology, Jagiellonian University Medical College, Kraków, Poland; b Division of Geriatrics, Department of Internal Medicine, Istanbul Medical Faculty, Istanbul University, Istanbul, Turkey; c Section of Geriatrics, Department of Internal Medicine, Faculty of Medicine and Health Sciences, Ege University, Izmir, Turkey; d Specialized Hospital for Geriatric and Palliative Medicine “13 November” Skopje, North Macedonia; e Medical Department, Emeis, Portugal; f First Department of Propaedeutic Internal Medicine, School of Medicine, National and Kapodistrian University of Athens, Athens, Greece; g Department of Clinical Sciences Helsingborg, Lund University, Helsingborg, Sweden; h Hospital CUF Descobertas, Lisbon, Portugal; i Preventive Medicine and Public Health Institute, Faculty of Medicine, University of Lisbon, Lisbon, Portugal; j Section of Geriatrics, Department of Internal Medicine and Paediatrics, Faculty of Medicine and Health Sciences, Ghent University, Ghent, Belgium; k Hellenic Society for the Study and Research of Aging, Greece; l LNA Santé, France

**Keywords:** Long-term care, Nutrition, Malnutrition, Geriatrics

## Abstract

**Objectives:**

High quality nutrition and medical nutrition therapy in long-term care (LTC) settings are essential to overcome malnutrition and ensure a better quality of life. Heterogenous and often insufficient regulations contribute to a disparity in nutritional practices across Europe. The aim of the study was to gather information on nutrition-related regulations and practices, as well as establish potential future directions in LTC settings in five European countries: Greece, North Macedonia, Poland, Portugal and Türkiye.

**Design:**

A country-specific literature review was conducted, including analysis of national regulations on nutrition in LTC settings. Additionally, the authors discussed possible future directions for each of the areas included in the review.

**Results:**

In all the countries included regulations regarding the nutrition of LTC residents were identified. Countries differed in the availability of nutritional guidelines for this specific population, the requirement to employ a dietitian, and the presence of a mandatory nutritional assessment. No country-specific tools were identified for screening, assessment and diagnosing malnutrition. Meals are adapted in all countries to accommodate religious and/or cultural dietary preferences.

**Conclusion:**

Areas for significant improvement were identified in all countries. The exchange of experiences between different European countries can lead to the development of good practices and a common direction for quality improvement regulations for nutritional care in LTC.

## Highlights

• Formal regulations regarding the nutritional approach to long-term care residents are insufficient.
• There is a lack of country-specific tools for screening, assessing and diagnosing malnutrition in long-term care settings.
• Engagement of all long-term care workers is needed to ensure that the nutritional needs of residents are met.
• Cultural and religious traditions were taken into consideration when designing menus for long-term care residents.

## 1 Introduction

According to the position paper of the International Working Group for Patients' Right to Nutritional Care, access to clinical nutrition is a human right because it lies between the right to food and the right to health, understood as the obligation to provide medical care in accordance with current medical knowledge and standards of care [1,2].

Nutritional care should be understood as an all-encompassing term for nutritional screening and assessment, individual nutritional requirements evaluation, design of meal plans, provision of meals, nutritional supplementation, and enteral and parenteral nutrition. In a broader view, it includes additional aspects such as oral health, swallowing assessments, social and financial aspects and functional assessments. It should also encompass the counselling and education delivered to patients and their caregivers, stakeholders, and the general public [3].

Long-term care (LTC) should include personal, social and medical services and ensure that people who are at risk of losing their autonomy maintain a level of functioning that is consistent with their fundamental rights, individual preferences and human dignity [4]. LTC covers both informal care, which in some cultural systems is the traditional model of care, where the responsibility lies mainly with family members, friends and neighbours, and formal care, which is delivered by paid healthcare professionals at nursing homes, residential care or in community settings, including the home [5]. LTC facilities are run by both public and private bodies and are highly heterogeneous across Europe in terms of the clinical complexity and functional status of residents. There is no uniform nomenclature to identify different types of LTC facilities across Europe, making comparisons difficult.

The aim of this study was to collect information on regulations and practices and discuss potential future directions for nutritional care in LTC settings in five European countries contributing to the PROGRAMMING 21122 COST Action (PROmoting GeRiAtric Medicine in countries where it is still eMergING): Greece, North Macedonia, Poland, Portugal and Türkiye.

## 2 Methods

The search on the respective national regulations and practices was carried out between March and July 2024 by researchers with expertise in long-term care of older people, nutritional care and geriatrics from countries with emerging/developing geriatric medicine: Greece, North Macedonia, Poland, Portugal, and Türkiye. Choosing those five countries among the 43 involved in PROGRAMMING COST Action was mostly a convenience sampling choice based on their eligibility to organise local events in the context of the Action’s activities to promote geriatric medicine (GM) across Europe through interactions with local stakeholders. All five of these countries are classified among those where GM is still emerging and are represented in the Action by reliable collaborators who agreed to contribute to this study.

The main areas to cover were regulations regarding nutritional care in LTC facilities of different European countries, practices in screening for malnutrition and nutritional assessment, organizational aspects of nutritional care including meal services (i.e., personnel, budget), and good practices regarding nutritional care in LTC, including projects, and campaigns.

The following search queries were used to search the PubMed, EMBASE and Web of Science databases:

(((“long-term care”) OR (LTC) OR (“long term care”) OR (“nursing home”) OR (“care home”) OR (“residential care”))) AND ((“nutritional care”) OR (“medical nutrition therapy”) OR (nutrition) OR (diet) OR (“nutritional assessment”) OR (malnutrition) OR (“enteral nutrition”) OR (“parenteral nutrition”) OR (“energy intake”))

In addition, researchers from the participating countries searched the country-level journals in their national languages as well as governmental websites and national repositories of legal acts using country-specific queries, as described below.

### 2.1 Greece

For Greek legislature, the website of The National Printing House of Greece (Greek: Eθνικό Tυπoγραφείo) [6] was searched, using the following query: “nutrition” AND (“nursing facility” or “older adult care home”) (Greek: διατροφή AND (νοσηλευτικό ίδρυμα OR μονάδα φροντίδας ηλικιωμένων). The search yielded 132 results which were manually checked by one author (EK). The accuracy of the results was confirmed by sending a written query to the board members of the Greek ESPEN chapter and the Greek Association of Older Adult Care Home Owners.

### 2.2 North Macedonia

For the search of the North Macedonian regulations, standards and legislature, the queries “nutrition in older adults / North Macedonia” OR “nutrition in LTC / North Macedonia” in English and “исхрана кај стари лица” OR “квалитет на исхрана во старски домови” OR “правилник за исхрана” in North Macedonian were used. The following websites and databases were searched: Ministry of Labour and Social Policy (North Macedonian: Mинистерство за труд и социјална политика) [7], Ministry of Health of the Republic of North Macedonia (North Macedonian: Mинистерство за здравство) [8], Official Gazette of the Republic of North Macedonia [9]. The data collection was performed by one researcher (BPZ) who consulted the North Macedonian Society for Nutrition and Health and the Institute of Public Health / Department of Nutrition for data accuracy.

### 2.3 Poland

For Polish legal acts, the Internet System of Legal Acts (Polish: Internetowy System Aktów Prawnych, ISAP) [10] was searched using the following predefined key words in separate search: “nursing facilities” (Polish: domy opieki) (26 results), “food and nutrition” (Polish: żywność i żywienie) (344 results), “healthcare” (Polish: opieka zdrowotna) (500 results). The same phrases were searched in LEX [11], the software gathering all polish legal acts and related documents. Additionally, the websites of the Ministry of Family, Labour and Social Policy [12], the Ministry of Health [13], and the Supreme Chamber of Control [14], Pubmed and Google Scholar were searched for documentation on standards and regulations regarding nutrition in LTC. The results were manually checked by two authors (AR, KP) and any concerns were resolved in discussion with KP.

### 2.4 Portugal

Two authors (AR, SD) performed the search in Portuguese databases. The following sources were searched: the Official Gazette (Portuguese: Diário da República) [15] for official legislation and regulations, the Nutritionists’ Association (Portuguese: Ordem dos Nutricionistas) [16] for professional guidelines and recommendations, Google Scholar: for literature relevant to nutrition practices in Portuguese long-term care settings. The following query was used for search: “Nutrition” OR “Nutritionist” AND “Residential Care Facilities for older persons” OR “ERPI (Portuguese acronym for the Residential Care Facilities for older persons)” OR “Residential care facilities” (Portuguese: “Nutrição” OR “Nutricionista” AND “Estruturas Residenciais para Pessoas Idosas” OR “ERPI” OR “LARES”)

### 2.5 Türkiye

For legislature from Türkiye, the Turkish Presidential Legislation Information System (Turkish: Cumhurbaşkanlığı Mevzuat Bilgi Sistemi) [17] was searched by four authors (SO, GO, SS, and GB) using the following query ‘nursing home’ OR ‘nursing homes’ OR ‘care home’ OR ‘nursing homes’ OR ‘home care’ (Turkish: “huzurevi” OR “huzurevleri” OR “bakımevi” OR “bakımevleri” OR “evde bakım”). For the extraction of standards, the official website of the Republic of Türkiye, Ministry of Family and Social Services, General Directorate of Services for Persons with Disabilities and the Elderly (Turkish: Türkiye Cumhuriyeti Aile ve Sosyal Hizmetler Bakanlığı Engelli ve Yaşlı Hizmetleri Genel Müdürlüğü) [18] was used.

## 3 Results

### 3.1 Dietary recommendations

All participating countries are legally obliged to provide all meals to residents. In North Macedonia, Portugal and Türkiye, it is recommended to follow the specific national dietary recommendations for LTC residents. In Greece and Poland, there are no dietary recommendations for institutionalised older adults.

In Greece, meals must meet a minimum of 1600 kcal/day and accommodate common co-morbidities, e.g. low-sodium, diabetes, etc., though there is no mandate for adjustments for dysphagia or sarcopenia. LTC facilities are not required to routinely report menus or nutritional status data of their residents to regulatory authorities.

In North Macedonia the primary regulation for nutrition in LTC is the *Rulebook on the Norms for All-Day Nutrition of Users in Institutions for Institutional Social Protection* [19]. It divides residents’ nutritional needs into two groups: active residents, requiring 8357 KJ (2000 kcal), and bedridden residents, requiring 7100 KJ (1700 kcal). Public LTC facilities must submit menu reports to the regional public health centre every three months. The Public Health Institute prepares reports on the quality of nutrition in the affected populations of the territory of the state annually. Diet quality assessment includes examining the energy and macro- and micronutrient content in food units consumed by the monitored population. Other bodies monitor biochemical and microbiological food safety in both public and private LTC facilities.

In Poland, there are no regular controls on served menu items, but there are two institutions authorised to check the biochemical safety of the food served (State Sanitary Inspectorate) and the standards for the use of public funds, including the quality of meals and conditions for serving meals (the Supreme Chamber of Control).

The Portuguese LTC legislation includes basic regulations on nutrition, requiring institutions to provide appropriate nutrition, adhering to medical prescriptions for residents [20]. A national reference manual for LTC facilities outlines healthy nutrition rules and their adaptations for specific diseases [21]. The food requirements of each facility must be reviewed every 3 months. The Directorate-General of Health provides meal guidelines for social support institutions that align with national food habits, and a proposal of a tool for qualitative evaluation of menus designed for older adults.

In Türkiye, different laws regulate LTC facilities, including nursing homes and adult day care/home care services. The Ministry of Family, Labour and Social Affairs issued a directive “Care Services Quality Standards” and a guide for its implementation covering caloric and fluid intake, feeding principles for residents with dysphagia, nutritional assessment, and medical nutrition therapy [22].

### 3.2 Meal costs

The costs of the meals provided in LTC settings varied between the participating countries.

In Greece, the average cost of each home-prepared meal per person is estimated at €2-€4; for meals ordered from an inexpensive restaurant, the average price is €15. The average pension in Greece is €720-€800, with a wide variability (€380-€2500). In North Macedonia there is no specific quota for daily meals in LTC institutions, although the Public Health Institution 13 November estimates price of daily cost for meals around 250,00 MKD (∼ €4) per resident. According to official data from the Pension Insurance Fund, the average pension in December 2023 was 20,195 MKD (€324), (€208 minimum pension - up to around €1000 maximum).

In Poland, there is no legal quota to be spent on meals in LTC institutions. An audit conducted by the Supreme Chamber of Control in 2010 revealed that the daily allowance for a patient's food in residential homes ranged from 3 PLN to 12 PLN (respectively €0.69 and €2.77); the average pension in Poland in 2010 was 1698.35 PLN (€390.68) [23,24].

In Portugal and Türkiye, there is no specific indication or regulation regarding the expenditure on meals within LTC institutions. The management of meal costs is determined individually by the institutions, regardless of whether they are public or private [25].

### 3.3 Public purse financing for Food for Specific Medical Purposes (FSMP), enteral (EN) and parenteral nutrition (PN)

In Greece, PN is provided in hospitals and nursing homes. It can then be continued and financially covered by the national healthcare provider while the patient recovers at home. EN is covered for patients with gastrointestinal diseases precluding oral feeding, as well as for patients with percutaneous gastrostomy or jejunostomy. Prescription of oral nutritional supplements (ONS) is restricted to cancer patients and those with ailments of the gastrointestinal tract leading to malnutrition documented by a specialist, but not for geriatric syndromes such as malnutrition or dysphagia in older adults.

In the North Macedonia, EN and PN is available and covered by health insurance only for hospitalised patients in acute hospital settings, rehabilitation facilities and the LTC Specialised Hospital 13 November. It is not covered for residents of public and private nursing homes. The use of ONS is not covered in any type of institution.

In Poland, EN and PN are funded by the state budget for residents of both residential and nursing homes. The use of ONS is not subsidised in either type of institution.

In Portugal, there is no state funding or reimbursement for ONS or EN, regardless of the indication.

In Türkiye, nutritional products may be covered by the public health system for certain indications specified in the report of the Health Board for persons with social insurance. Oral and EN products are reimbursed in case of cancer and gastrostomy, without the need for a diagnosis of malnutrition. Apart from these cases, these products may also be reimbursed for certain conditions associated with malnutrition (e.g. dementia, chronic kidney disease, exocrine pancreatic disorders). For patients who cannot be fed orally or enterally, PN is covered.

### 3.4 Nutritional assessment

In North Macedonia, Portugal, and Poland, there is no legal requirement for nutritional assessment upon LTC facility admission or during the stay. In Poland, only the dependency assessment of the patient being admitted to nursing home using the Barthel Index [26] is obligatory, while in North Macedonia the Barthel scale is used selectively. However, some LTC facilities, particularly nursing homes with medical services, do conduct nutritional assessments in practice.

In Greece, a new mandate to perform a nutritional risk assessment within 24 h of admission was introduced into law in July 2024, applicable to all hospital and nursing facilities. The regulation requires that patients at moderate or high nutritional risk receive an individualised nutritional plan within 24 h of the assessment, prepared by the unit's nutrition support team (consisting of a nutritionist, a physician and a nurse). Low risk patients should be reassessed on day 7 of their stay [27]. Popular instruments to assess and monitor nutritional status of older residents in Greek hospitals and LTC facilities are the Mini Nutritional Assessment (MNA) [28], MNA-SF (short form) [29], or the Geriatric Nutritional Risk Index (GNRI) [30]; similarly, as mentioned above, some scales including the Barthel Index are used to evaluate dependency.

In Poland, the tools that might be used include the MNA, that was validated in the Polish population of older adults in LTC settings [31], and the Subjective Global Assessment (SGA) [32]. In many residential and nursing homes, residents are weighed once a week or when medically indicated, but this is not mandatory. A full nutritional assessment with laboratory blood tests is required for residents starting enteral or parenteral nutrition.

In Portugal and Türkiye, the use of the MNA is recommended for assessment within 48 h of a resident's admission, after significant events (e.g. hospitalization, infections), and every 3 months [22,33,34]. Additionally, in Türkiye, residents must have their body weight, or its equivalent (calf or arm circumference) measured every two months [22].

### 3.5 Tools for intake assessment

In all participating countries there are no tools for monitoring caloric and fluid intake other than descriptive notes made by nursing staff. Recording fluid and meal quantities is usually done only for patients with feeding tubes. In Portugal and Türkiye, it is recommended to keep and analyse daily dietary records of residents, but there are no specific protocols on how to do this and there is no legal requirement. None of the countries used artificial intelligence assisted meal tracking nor other forms of automated intake monitoring.

### 3.6 Availability of culturally adapted food and nutrition in LTC

Adherence to religious dietary rules includes beneficial practices, such as abstaining from alcohol. However, other practices, such as prolonged fasting, may lead to deficiencies in essential nutrients, such as calcium, iron, vitamin D, and vitamin B12 [35].

In Greece, over 90% of the population is Christian Orthodox [36], thus all hospitals and LTC facilities provide an option of vegetarian meals during the 40-day religious fast of meat preceding Passover.

The population of North Macedonia is religiously mixed, with approximately 80% Christians and 20% Muslims [37]. LTC facilities are not obliged to follow cultural or religious requirements regarding meals, but individual requests are always respected.

In 2021, more than 70% of Polish adults claimed to be Catholic [38]. There is a tradition in Poland of not eating meat (except fish) on Fridays for religious reasons. Therefore, LTC facilities often offer vegetarian or pescatarian meals on Fridays. Similarly, in Portugal, 80% of the population aged 15 and older identifies as Catholic according to the 2021 census [39]. Many LTC centres are run by religious institutions and follow catholic traditions, including ceremonies on Sundays, and dietary practices such as abstaining from meat on Good Friday.

In Türkiye, for residents who prefer to fast during Ramadan, meals are served twice a day: during Suhoor (i.e. the meal before sunrise before fasting) and Iftar (i.e. the meal to break the fast at sunset). Snacks are also served in the interval between Iftar and Suhoor for residents who prefer fasting. On the first day of Eid al-Adha, many LTC facilities serve meat products for breakfast or one of the other main meals. Desserts are served to residents on the first day of the festival. As most residents are Muslim, pork products are not included in any meal in LTC facilities in Türkiye. Care is taken to ensure that meat products are slaughtered according to Islamic methods.

Although the older person’s preferences and values predominate in health-related decisions, some considerations may arise regarding the adequacy of nutritional and hydration intake during fasting periods. In none of the countries included were we able to identify any regulations regarding the calorie and protein content of meals adapted for religious reasons. This may be particularly important during long periods of fasting such as Ramadan or Lent.

### 3.7 Personnel responsible for nutritional care

The status of the dietitian profession varies across the participating countries, with some having a protected title whereas in others it remains under-recognised. It is common for nutrition-related tasks to be shared between different health professionals in LTC settings.

In Greece, hospitals are required to have a Department of Nutrition, manned by at least 2 professionals specialized in nutrition and dietetics. Each specialized professional can follow up to 80 patients [40]. LTC facilities are encouraged, but not required, to employ a nutritionist, and nutritional care is often undertaken by nurses and visiting physicians.

In North Macedonia mostly physicians (who perform nutritional assessments and provide nutritional treatment) and specialized nurses (who perform nutritional interventions and education) are responsible for the process of nutritional care. Trained caregivers perform assisted feeding.

Similarly in Poland, the competencies related to the provision of nutritional care are shared among different healthcare professionals. In some nursing homes there are nutrition care teams usually consisting of a dietitian, a physician, and a nurse responsible for providing and supervising enteral or parenteral nutrition.

In Portugal, since the employment of a doctor or a nutritionist is not mandated by current legislation, nutritional care in LTC facilities is typically managed by nurses, cooks, and trained caregivers. The Professional Regulatory Body of Nutritionists recommends the presence of nutritionists in multidisciplinary care teams [41].

The nutritional care in LTC settings in Türkiye is performed within a team, including dietitians (responsible for nutritional programmes and assessment of nutritional status and intake), doctors (nutritional assessment if a dietitian is not available, identification of special dietary needs), nurses (supervision of the distribution of meals, intake assessment, assistance in eating), older adult care personnel/technicians (assistance in eating, practical implementation of nutritional care instructions), and facility managers [22,42].

The competencies associated with the provision of elements of nutritional care by different health professionals within a multidisciplinary team are presented in the supplementary material (Fig. S1, in supplementary material).

### 3.8 Availability of programmes aimed at improving the quality of nutritional care

Measures to improve the quality of nutritional care can be divided into those related to raising public awareness and those related to strengthening the control of nutritional practices in LTC facilities.

In Greece, North Macedonia and Poland, there are no dedicated governmental programmes aimed at improving the quality of nutritional care delivered in LTC institutions. There are supervisory authorities that share responsibilities for monitoring the quality of medical and nutritional services. In Poland, the Supreme Chamber of Control carried out audits on the functioning of care institutions (2010) and on the availability of LTC services funded by the national health system (2020) [24,43].

In Portugal, there are no systematic government programmes aimed at improving the quality of nutritional care delivered in LTC settings. The Economic and Food Safety Authority is the entity responsible for overseeing food safety, but its inspections are typically reactive, responding to complaints or issues [44,45]

In Türkiye, important documents regarding nutrition were published, including “*The Türkiye Healthy Aging Action Plan and Implementation Programme 2021-2026*”, aimed at malnutrition prevention among older adults living in the community and in LTC facilities [46].

In recent years, there have been efforts to increase awareness of the public on the topics of malnutrition and sarcopenia through advertisements in the press and media, mainly driven by academic research initiatives, non-governmental organizations, and the private sector. Countries and organisations with an interest in nutrition use different types of strategies to promote topics related to the importance of nutrition in the management of chronic diseases and its role for patients in LTC facilities. In addition to governmental programmes, there are campaigns that aim to raise awareness of malnutrition and the importance of good nutrition in LTC settings. One of these is NutritionDay (nDay) Worldwide - a campaign aimed at raising awareness of malnutrition in different settings, including hospitals, intensive care units, primary care facilities and LTC facilities [47]. Of the countries involved in this study, Greece, Poland, Portugal and Turkiye are participating in the nDay initiative. Additionally, in Türkiye the “Nutrition Academy-Nursing Home” was launched, aiming to raise awareness and provide training on nutrition-related topics among healthcare workers [48].

## 4 Discussion

Having reviewed nutritional practices and regulations in five different European countries, we revealed a huge legislation gap for nutrition-related topics in LTC. National nutrition recommendations for long-term care facilities were found in North Macedonia, Portugal and Türkiye. We demonstrated that none of the analysed countries had established legally required meal cost quotas for residents' meals. There was a wide variation between countries in terms of public purse financing for medical nutrition, ranging from no state funding in North Macedonia and Portugal to full coverage in Türkiye. Only Greece implemented the full nutritional assessment procedure for hospitalised patients within the regulations. All of the included countries lacked tools for intake assessment. Recommendations to employ a dietitian in long-term care (LTC) facilities were found in Greece, Portugal and Türkiye, while in North Macedonia and Poland, nutrition-related responsibilities are shared between healthcare professionals, and there is no requirement or recommendation to employ a dietitian.

**Fig. 1:**
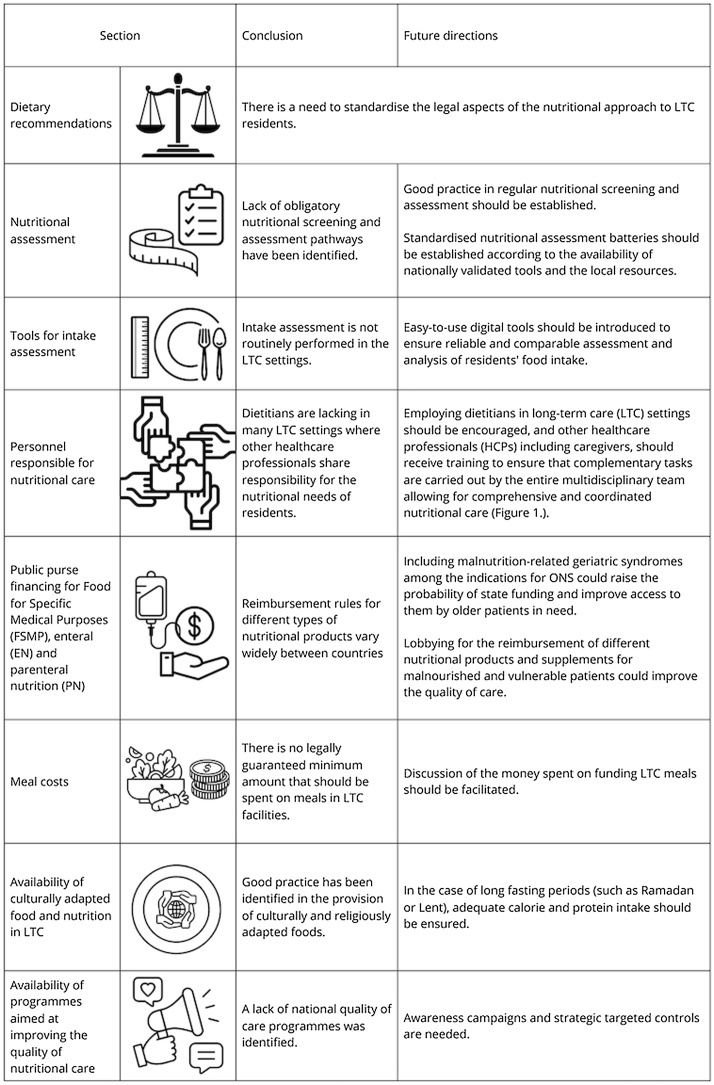
Conclusions and future directions for nutrition-related care practices in long-term care setting.

Following an evaluation of Greece, North Macedonia, Poland, Portugal and Türkiye's nutritional practices and regulations, we have formulated a summary of the present state and future directions for nutrition in long-term care facilities (Fig. 1).

The lack of standards affects nutritional assessment and intake monitoring, meal composition and their costs, reimbursement possibilities for food for specific medical purposes and enteral nutrition. In the World Health Organization (WHO) document ‘*Rebuilding for sustainability and resilience: Strengthening the integrated delivery of long-term care in the European Union*’, the experts summarise the objectives of formal LTC and future directions for the formalised provision of care services [4]. According to the WHO, the role of long-term care services lies between the health care system and the social protection system, sharing some of the challenges, such as disease management, health promotion and prevention of social exclusion. The document promotes the following principles of LTC systems: person-centeredness, user involvement, prevention, healthy aging and enablement [4]. The realisation of these qualities and tasks takes place on several levels, including the provision of adequate and state-of-the-art medical services for LTC residents, appropriate living conditions and measures to activate and accommodate the individual's deficits. It is also important to provide access to proper, health-promoting nutrition, as it is crucial for disease management and health promotion. In our manuscript, we presented some significant findings at system level. However, it is also important to consider the actions that can be taken at the institutional level to greatly impact individual residents’ quality of life. According to Keller et al., LTC institutions can be used for reframing evidence-based nutrition practices into relationship-centred care. As good quality nutrition is closely linked to building and maintaining social contact, this can be seen as one of the most important factors promoting healthy ageing and nutritional therapy [49]. The opportunity to eat together remains in line with the WHO LTC principles mentioned above, enabling residents’ activization and promoting community interactions [4]. Employees of LTC institutions are primarily responsible for implementing these changes. It is therefore important not only to raise their awareness of the importance of proper nutrition for the health and wellbeing of older adults, but also to make them more sensitive to the difficulties they may experience with food and eating in general.

A pro-quality direction that can ensure better nutritional care for LTC residents is to take steps towards greater regulation of nutritional solutions and the establishment of bodies to monitor institutions’ compliance. Recommendations from scientific and professional societies can serve as a foundation for publishing regulatory documents, thereby establishing nutritional interventions as the standard of care rather than an option.

Good practices can also be drawn from countries outside Europe. In 2022, Dietitians of Canada, a nationwide organisation for dietitians, published a set of standards for long-term care nutrition and food services that align with many of the areas we identified as important based on a literature review [50]. In February 2025, the Australian government's Department of Health and Aged Care published the Strengthened Aged Care Quality Standards, which included a section on food and nutrition, for public consultation [51]. Despite their differing cultural backgrounds, health care systems and policy contexts, these documents could set as valuable reference and direction for future development.

There is a visible lack of guidelines for standardized, repeatable nutritional assessment in most countries included in the study. Establishing clear practices for assessing nutritional status is a necessary basis of care with core elements universally applied, and culturally sensitive adaptations as needed. Assessment of nutritional status should include objective, easy-to-perform anthropometric measurements and validated screening scales so that the process can be repeated and compared over time [52]. Proposed assessment tools should be structured, repeatable, linguistically and culturally validated for the given population and should not add to the workload burden of care providers. In their proposed standards, Dietitians of Canada [50] recommend that assessments should include nutritional status, food preferences, the ability to eat independently, and a swallowing assessment. Additionally, the Australian standards [51] also pay attention to what constitutes a positive dining experience for the patient. Solutions using new technologies, photo analysis and artificial intelligence, which are currently receiving a lot of attention, could be used in the future to assess intake in LTC settings [53,54]. There is a need to fund grant programmes aimed at finding digital solutions for LTC systems. Healthcare workers should be trained on the importance of overall nutritional care, and the lower level of development of geriatric medicine in several European countries may be an additional obstacle [55]. Other than advocating for the development of good clinical practices on the care of older people across Europe, the promotion of education of the whole workforce in basic principles of GM is required [56].

In the countries included in our research, there was no established quota to be spent on meals in LTC facilities. A reasonable budget allocation should be ensured to provide nutritious meals tailored to the individual nutritional needs of each resident. We noticed that the quotas allocated for meal costs were relatively low, thus periodic controls on the quality and quantity of the products supplied should therefore be mandatory [57,58]. It may be recommended to seek less expensive alternative food products (through the use of locally sourced food products for example) that still have the required nutritional value and quality to ensure both a healthy diet and ensure the sustainability of LTC facilities. The financial aspect of nutritional care encompasses not only traditional meals, but also medical nutrition therapy products, such as oral nutrition supplements and enteral nutrition. As for traditional meals, validation of a Foodservice Costing Tool (FCT) to assist with nutrition planning in LTC facilities is underway [59]. The authors of the tool highlight various relevant costs, such as food, labour and utilities, involved in providing food services to residents. Regarding medical nutrition therapy, reimbursement for different types of nutritional products varies between countries. Malnutrition is related to greater financial costs, as malnourished residents are more prone to illness and require more medical consultations and medication. In a study of the cost of malnutrition in Dutch nursing homes, the authors found that while the regular cost of nutritional care in long-term care facilities is 319 million euros per year, the financial burden associated with managing malnutrition is almost twice as high [60]. According to a study by Elia et al., the use of oral nutritional supplements may be a cost-effective intervention to ensure a better quality of life for nursing home residents [61]. In a review by Hugo et al., environmental and food-based nutritional interventions were also shown to be a cost-effective way to improve malnutrition-related clinical outcomes and thus resident health in LTC settings [62].

We also identified good practices that support residents in observing their traditions and religious beliefs by offering culturally appropriate meal options. Due to the strong beliefs associated with the Christian and Islamic faiths, meals in all countries should be adapted according to religious preferences. However, in most countries there are no legal requirements nor attempts to ensure adequate provision of different nutrients within such meals. This is significant as these meals usually require restrictions on meat, which can contribute to protein deficiencies in the diet.

We discovered that a significant portion of nutrition-related tasks in LTC settings are carried out by health professionals other than dietitians. Increasing the employment of dietitians, while establishing and highlighting their role in LTC facilities could have a positive impact on the nutritional status of residents and reduce the burden of care of other health professionals. Alternatively, the provision of educational programmes and courses for healthcare professionals other than dietitians on the principles of healthy nutrition in old age, malnutrition prevention and treatment strategies could improve the quality of nutritional care in areas and facilities where it is currently not possible to employ a specialist. A group that should be considered when designing educational materials are the professional caregivers employed in LTC facilities. Their qualifications differ from those of other healthcare professionals, and in many LTC facilities they often spend a significant amount of time with patients during the day, assisting them with eating and knowing their individual preferences and eating habits [2]. Raising their awareness of the importance of malnutrition and increasing their ability to recognise its symptoms can support medical staff in diagnosis, and subsequent initiation and monitoring of therapeutic interventions. Acknowledging this need, the consensus on training curricula for non-specialists’ healthcare professionals on basic concepts of geriatric care is the main goal of the PROGRAMMING COST Action. According to preliminary PROGRAMMING research presented at the 20th EuGMS congress 2024, except for nutritionists, nurses and dentists, education on malnutrition and sarcopenia is not mandated as a part of the undergraduate curriculum by the pan-European professional societies of other geriatric interdisciplinary team members (i.e. physical therapists, occupational therapists, speech–language pathologists, clinical pharmacists, psychologists) [63]. To investigate healthcare professionals’ educational needs on nutritional care, nutrition-related topics were included in an online survey of educational needs the Action launched in order to capture the real needs of stakeholders in various fields. Healthcare professionals were asked to report their perceived current knowledge, relevance to their clinical practice and interest for further education and training on various topics related to geriatric medicine, including sarcopenia and nutritional assessment and management of malnutrition of the older person, including: (1) identifying older people who are malnourished or at risk of malnutrition using the Mini Nutritional Assessment – Short Form or other screening tools and (2) knowing when to refer to a dietitian or when to prescribe nutritional supplements [64]. Additionally, focus groups among healthcare professionals have been also conducted, including personnel working in LTC settings. Results of these educational gaps and needs’ assessment are currently under analysis and will be communicated in a separate publication.

Training in nutrition-related domains would also facilitate an informed multidisciplinary nutrition care process and enable non-dietitian providers to be fully aware of the importance of proper nutrition for the health status of residents. The aspects that we propose as important for all LTC workers are core principles concerning the nutritional status of patients (nutritional screening and assessment, providing medical nutrition therapy, monitoring the effects of such actions) as well as topics related to feeding patients (intake monitoring, safety during the feeding process, first aid in the event of aspiration).

None of the countries included mentioned having structured programmes for teaching such skills. The nutritional knowledge of LTC healthcare workers from different professional backgrounds is usually acquired during their formal training and may not have been updated for years. As financial constraints very often do not allow for the fulltime employment of a dietitian in every LTC facility, there should be an opportunity to use training provided by community dietitians for healthcare staff in nursing and care homes. Other types of training, such as online courses, can also be used to ensure a higher level of awareness of nutritional challenges in LTC settings. PROGRAMMING has dedicated a whole Working Group to the framework of training methods.

In general, there are not many actions and projects that focus on the nutrition of older adults in LTC settings. One of the reasons may be due to a common misconception that since they are institutionally supplied with meals, residents do not need to be educated about the importance of nutrition in old age. However, it appears that institutionalised older adults can benefit from educational services for expanding nutrition-related health literacy adapted to their needs and level of understanding [65]. In the absence of tools to monitor intake or common practices to assess nutritional status, it seems reasonable to raise awareness of malnutrition among residents so that they can advocate for themselves in accessing appropriate quality nutrition and medical care for weight and appetite loss. Another important direction for nutritional education is the training of health professionals. All five countries participating in this research are part of the PROGRAMMING (PROmoting GeRiAtric Medicine in countries where it is still eMergING) COST Action, which was launched in 2022. As one of the main objectives of PROGRAMMING CA 21122 is to establish a formal framework for the training of physicians and other health professionals without prior training in geriatric medicine, the Action is expected to contribute significantly to the future development of geriatric medicine in all settings, including LTC environments [66]. To close this gap, coordinated action by policymakers, researchers, and LTC providers is urgent and overdue.

### 4.1 Limitations

While important, our study is not without limitations. Firstly, due to the study design, we include only five countries with distinct cultural and historical backgrounds, and varying degrees of development in geriatric medicine, which limits the ability to extrapolate the findings on countries with well-developed GM. Furthermore, due to a lack of legislation or a small number of studies addressing nutrition-related issues in the LTC setting within certain countries, we had to partially rely on the authors' clinical experience and policy knowledge when describing the state of nutrition in LTC.

## 5 Conclusions

The management and coordination of nutritional services in LTC requires the development and implementation of effective policies and best practices for providing adequate nutritional care that meets the individual needs of residents. The current practices and regulations identified in the five countries included in the study do not allow the needs of older residents to be fully met and represent a challenge for governmental, scientific and organisational bodies.

We identified the following directions as urgent to ensure better quality of nutritional practice in LTC settings in countries where geriatric medicine is still developing:

• Integrating nutritional care in national policies aiming to improve the health of older people and tackle geriatric syndromes
• Include geriatricians in the strategic planning of national health policies
• Establish national regulations and monitoring mechanisms for LTC facilities that highlight comprehensive nutritional care as a core responsibility towards residents and a quality evaluation index for facilities
• Encourage research teams and academia to translate and validate screening and assessment tools for malnutrition
• Urge policymakers to seek out international standards on LTC nutrition from countries where they are well established
• Encourage academia to translate international guidelines and/or national scientific societies to produce their own, culturally adapted, nutrition-related guidelines, including guidances for LTC
• Invest in research, new technologies and telemedicine possibilities in order to optimise the use of limited resources and scarce expertise
• Conduct cost-effectiveness studies regarding the cost of malnutrition in LTC settings
• Advocate the importance of identifying malnutrition in LTC settings and the benefits of its prevention and management to policy makers and facility managers
• Form national working groups to adapt internationally available nutritional recommendations for LTC to local circumstances, cultural and religious specificities
• Promote the education and training of malnutrition of older adults across all levels of education of healthcare practitioners
• Organise campaigns to raise public and end user awareness

PROGRAMMING proposes education and training of healthcare professionals working in LTC settings as an initial pragmatic solution to tackle these challenges, but policymaker engagement and governmental regulations at a national level are a crucial step to further advance quality care for older people, especially nutritional care.

## ORCID ID

Anna Rudzińska: 0000-0002-8369-2131

Gulistan Bahat: 0000-0001-5343-9795

Evrydiki Kravvariti: 0000-0003-4330-3266

Karolina Piotrowicz: 0000-0002-4760-8588

## Declaration of Generative AI and AI-assisted technologies in the writing process

The authors used AI-assisted technologies to improve readability and language of the work.

## Declaration of competing interest

The authors declare that they have no known competing financial interests or personal relationships that could have appeared to influence the work reported in this paper.
