# Supplementary material for: Nutritional practices in long-term care across five European countries: Findings from the COST Action PROGRAMMING
Source: J Nutr Health Aging. 2025 Aug 26;29(10):100650. doi: 10.1016/j.jnha.2025.100650 (PMC12789832; doi:10.1016/j.jnha.2025.100650)
Supplement: Supplementary file 1 [file mmc1.docx]

|  | Greece | North Macedonia | Poland | Portugal | Türkiye |
| --- | --- | --- | --- | --- | --- |
| Legislation aspects N | No specific rules. | National recommendations. | No specific rules. | National recommendations. | National recommendations. |
| Meal costs | No established legal meal cost quota | | | | |
| Public purse financing for Food for Specific Medical Purposes (FSMP), enteral (EN) and parenteral nutrition (PN) | FSMP not covered for indications related to older age | No state funding. | The use of FSMP is not covered. | No state funding. | Fully covered. |
| Nutritional assessment | Mandatory assessment | No legal requirement for nutritional assessment upon LTC admission or during the stay | | Recommendation to screen | Recommendation to screen |
| Tools for intake assessment | No mandatory tools for intake monitoring | | | Recommendation to track daily dietary intake of residents, but no legal requirement | |
| Availability of culturally adapted food and nutrition in LTC | There is no legal requirement, but residents' wishes are respected. | | | | |
| Personnel responsible for nutritional care | Recommendation to employ nutritionist | Nutrition-related responsibilities are shared between healthcare professionals, no requirement nor recommendation to employ dietitian | | Recommendation to employ nutritionist | Recommendation to employ nutritionist |
| Availability of programmes aimed at improving the quality of nutritional care | No dedicated governmental programmes aimed at improving the quality of nutritional care in LTC | | | | Governmental programmes aimed at LTC facilities |
