# Supplementary material for: Nutritional practices in long-term care across five European countries: Findings from the COST Action PROGRAMMING
Source: J Nutr Health Aging. 2025 Aug 26;29(10):100650. doi: 10.1016/j.jnha.2025.100650 (PMC12789832; doi:10.1016/j.jnha.2025.100650)
Supplement: Supplementary file 2 [file mmc2.pdf]

Nutrition care pathway in LTC facilities

MULTIDISCIPLINARY NUTRITIONAL CARE TEAM

Doctors | Nurses | Nutritionists | Managers | Social Workers | Physiotherapists | Occupational Therapists | Speech therapists | Dentists | Cooks | Helpers | Pharmacists

ORGANIZATIONAL (M, Nt, MD, N, ST, PT, OT , SW, C, P)

- Design, coordination and monitoring of policy of nutritional care and protocols
- Nutrition related budget calculation (personnel, meals, facilities, equipment, medical nutritional therapy)
- Management and control of food supply, storage and preservation
- Monitoring of food and meals hygiene, quality and safety
- Establishment of basic standard nutritional recommendations
- Menu planning, including special diets
- Service of meals cooking or meals delivery by external provider
- Planning and organization of common dining areas and portable equipment for room meals
- MDT education on nutrition of older persons
- Delivering nutritional literacy for patients, families, and caregivers – standard nutritional recommendations, nutritional problems and its management

PATIENT TAILORED NUTRITIONAL CARE JOURNEY

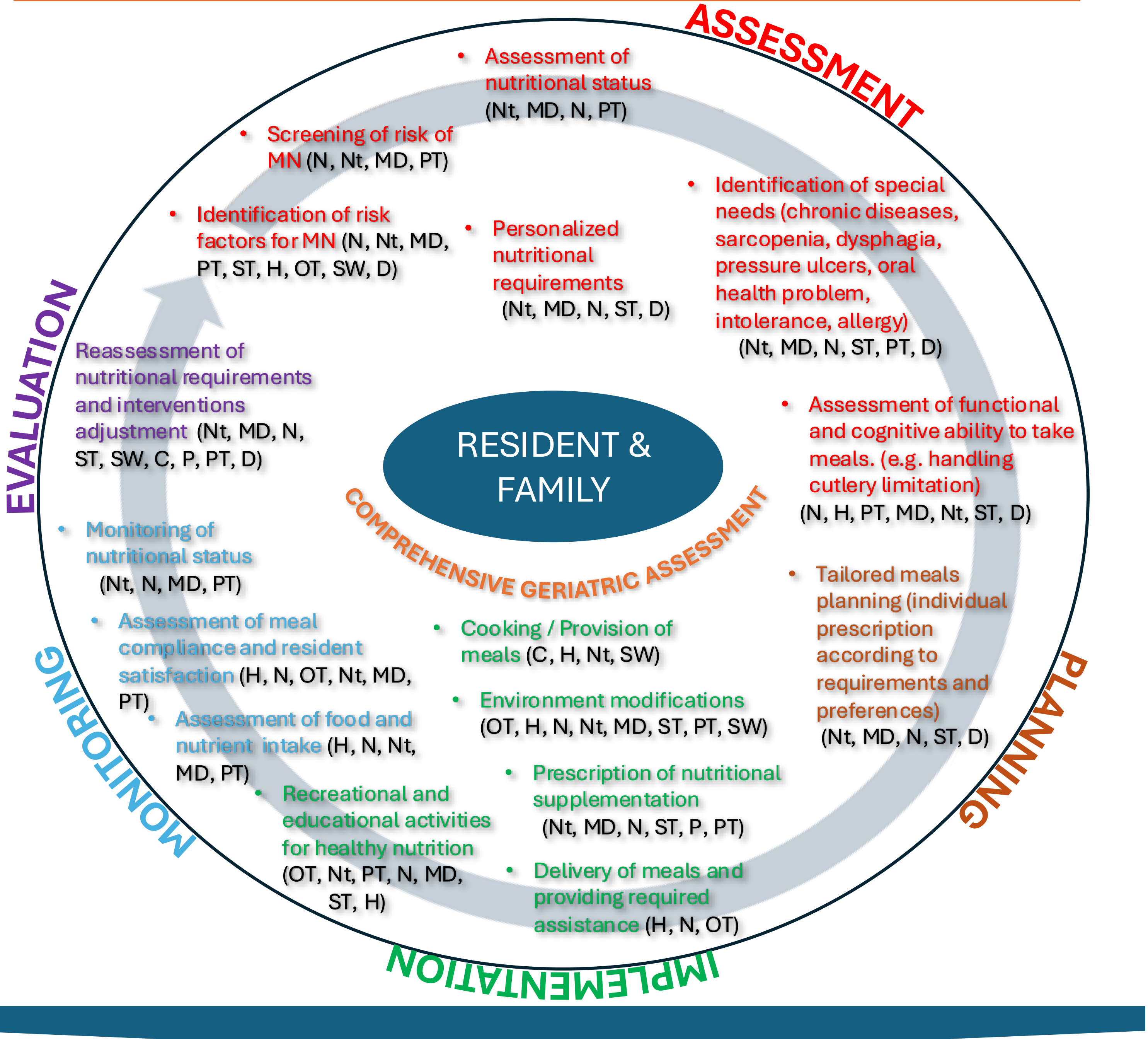

Maintenance of functionality, physical activity and cognition
Improvement of Quality of life

Roles and Responsibilities of LTC Multidisciplinary Team Members in the Scope of Nutritional Care

Administrators / Managers

- Role: Oversee the overall operation and administration of the nutritional care pathway
- Responsibilities: Ensure compliance with regulatory and quality standards, warrant sustainability, provide resources and support for the nutritional care team, and facilitate communication among departments.

Dietitian/Nutritionist

- Role: Provides expert nutritional advice for standard practices and participates in individual nutritional assessment and intervention
- Responsibilities: Assess of residents' nutritional status and nutritional requirements. Assessment of special nutrition requirements. Monitor of food preparation and cooking. Control of the quality of meals delivered by outsourced providers. Develop and implement standard and individualized nutrition care plans. Selection of preferrable menus for each resident, respecting cultural and religious habits and preferences. Prescription of nutritional supplements. Follow up on residents' nutritional status and adjust nutritional interventions as needed. Educate staff, residents, and families about adequate nutrition.

Nurse

- Role: Implement and monitor the nutrition care plan and residents' follow up on a daily basis.
- Responsibilities: Malnutrition screening and identification of risk factors. Assessment of the need for feeding assistance. Identification of special nutritional needs. Assist with feeding and nutritional supplements intake as needed. Monitoring residents' food and fluid intake, compliance and satisfaction with the meals plan and nutritional parameters. Ensure proper registry of nutritional intake. Notification of changes in residents' eating habits or health status to the dietitian and medical doctor. Training and supervising helpers / care assistants to assist in feeding according individual needs. Educate staff, residents, and families about adequate nutrition.

Medical Doctor

- Role: Diagnose and coordinate multidisciplinary interventions for nutritional problems and medical conditions and geriatric syndromes that can affect nutritional status. Provides expert nutritional advice for standard practices.
- Responsibilities: Comprehensive medical assessment, including diagnosing nutritional problems and other conditions that impact nutritional status and/or require special nutritional interventions. Prioritize clinical outcomes and nutritional interventions to achieve it. Prescribe therapeutic diets and nutritional supplements. Prescribe artificial nutrition. Follow up residents' nutritional intake reports and nutritional status. Educate staff, residents, and families about adequate nutrition. Coordinating the MDT team and ensuring an individualize, continued and

Helpers | (Nurse) Care Assistants

- Role: Deliver the meals and provide assistance to the residents on a daily basis.
- Responsibilities: Delivering the meals and ensuring their conditions to be taken. Ensure appropriate residents and environment conditions during the meals. Assisting with feeding as needed, promoting residents' autonomy as much as possible. Monitoring residents' food and fluid intake and compliance with the meals plan. Ensure proper registry of nutritional intake. Reporting changes in residents' eating habits or health status to the nurse. Reporting residents' preferences and satisfaction.

Speech and Language Therapists

- Role: Assessment of residents with swallowing, feeding problems and communication disorders and implementation of specialized intervention to ensure safe and effective feeding, swallowing and communication. Provides expert swallowing advice for standard practices.
- Responsibilities: Performing clinical swallowing evaluations and instrumental assessment, if needed together with otolaryngologist specialist. Identifying risk factors for aspiration. Assessing motor skills, cognitive and sensory functions affecting swallowing. Recommending appropriate dietary modifications (e.g. texture or consistency modified diets). Providing speech therapy to improve swallowing function. Advising nurses and helpers on feeding techniques, positioning, and strategies that facilitate safe eating. Collaborating with dietitians, cookers and nurses to integrate swallowing and feeding modifications into the resident's nutritional plan. Teaching residents to overcome communication barriers related to eating and encourage them to express any discomfort or issues related to food intake. Follow up residents' ability to eat and swallow safely and assess for changes in condition. Educate staff, residents, and families about safe feeding practices, appropriate techniques for supporting eating, and signs of aspiration or choking.

Roles and Responsibilities of LTC Multidisciplinary Team Members in the Scope of Nutritional Care

Occupational Therapist

- Role: Ensure maintenance or regaining skills needed for safe and effective feeding
- Responsibilities: Assessing residents' physical and functional ability to feed autonomously. Assessing environmental factor that can influence eating ability. Identifying barriers to meal participation (e.g. physical limitations, inadequate posture, cognitive and emotional problems). Providing adaptive equipment and training to enhance independence during meals. Recommending environment modifications and socializing opportunities. Develop strategies to improve fine motor skills and memory or attention issues that impact feeding. Teaching residents techniques to improve posture and using assistive devices during meals. Organizing meal/nutrition related activities to increase nutrition awareness. Educating staff on techniques that promote residents feeding autonomy.

Social Workers

- Role: Supporting social, cultural and financial aspects related to nutrition.
- Responsibilities: Assessing social, cultural and financial factors affecting nutrition (e.g. social isolation, loneliness, religious nutritional habits, poverty). Provide counselling and support to residents, families and other MDT members to ensure that social, cultural and financial aspects are respected and incorporated in the personalized nutritional plan. Coordination with community resources for additional support. Activation of social and financial benefits.

Cooks

- Role: Preparation and delivery of meals following nutritional care plans delivered by nutritionists.
- Responsibilities: Planning, preparing and cooking the daily menus, to meet dietary and treatment requirements and preserve the nutritional content of food. Planning provision of food and oversee food and kitchen conditions to ensure safety and quality standards. Following food storage, handling and hygiene practices. Assessment of residents' food and meals preferences, cultural, religious, or ethnic dietary practices, and aligning with nutritional requirements. Ensuring meals are attractive and appealing. Training kitchen staff on healthy cooking, special dietary needs and modifications (e.g. texture, ingredients).

Pharmacist

- Role: Management of medication therapy and its impact on nutrition.
- Responsibilities: Reviewing medication and identifying and monitoring drug-nutrients / foods interactions, including (over-the-counter) supplements. Selecting the best drug, dosage, schedule and formulation to minimize drug-nutrients interactions. Recommending deprescription of drugs that lead to anorexia, impact absorption and metabolism of nutrients, and lead to other nutritional symptoms and problems impacting nutritional status, if safer alternatives are available. Recommending nutritionists and cooks meals modifications that avoid impact on drugs absorption, metabolism and effect. Recommending appropriate nutritional supplements, correct dose and schedule. Coordinating medication schedule and meals and foods. Educating staff on medication-related nutritional problems.

Physiotherapists

- Role: Supporting the maintenance of physical function and mobility to ensure optimal nutritional intake and assist residents in eating independently and safely.
- Responsibilities: Assessing physical function related to eating, including strength, dexterity, swallowing and mobility. Recommend posture and positioning techniques for safe and effective eating. Supporting residents with mobility challenges to maintain independence during mealtimes e.g. recommending mobility aids. Developing exercise programs to improve hand strength, endurance for eating and increasing appetite. Train staff and caregivers to assist residents with physical needs during meals. Monitoring residents at risk of malnutrition due to physical limitations affecting eating. Coordinating with the MDT to align physical interventions with dietary plans.

Dentists

- Role: Ensuring the oral health of residents, which directly impacts their ability to eat, chew, and enjoy meals.
- Responsibilities: Assessing and treating oral health issues, including tooth decay, gum disease, and candidiasis. Ensure that dentures or prosthetics are functional and comfortable for efficient chewing. Advising on oral hygiene practices to prevent infections and maintain good oral health. Identifying and managing oral conditions that may impact food intake, such as dry mouth or pain. Recommending fluoride treatments or other dental interventions. Assisting in the prevention and management of mouth sores, which can affect eating. Collaborating with dietitians and cooks to recommend suitable food textures for residents with dental issues. Monitoring and treating any oral conditions that could contribute to malnutrition. Educating residents, staff and caregivers on the importance of oral health in nutritional care.
